# “The Right Stuff”: The Global Burden of Disease

**DOI:** 10.1371/journal.pmed.0040084

**Published:** 2007-02-27

**Authors:** Bolajoko O Olusanya

*PLoS Medicine* is one of the few journals with a dedicated forum for neglected diseases, and the suggestion by its editors that journals should give preference to diseases based on their relative contributions to the global burden of disease is noteworthy as a positive step towards optimising the global research agenda [1]. Undoubtedly, the recent report by Mathers and Loncar, like earlier versions of the Global Burden of Disease (GBD) study, represents the best effort yet at providing a level playing field for diverse diseases and health conditions globally [2]. A key feature of this project is the evaluation of health outcomes in terms of mortality and burden of disease indexed by disability-adjusted life years (DALYs). However, a few concerns still linger on the application and current scope of the study which have significant implications for health-care policy, particularly in the developing world.

The concept of the “burden of disease” was introduced to redress the inequality and inequity occasioned by the exclusive use of mortality as the summary measure of population health [3,4]. The DALY was thus intended to provide information on non-fatal health outcomes of diseases that have been largely neglected in health planning because of the “conceptual and definitional complexity of measuring morbidity and disability in populations” [5]. However, it is uncertain how far the well-intentioned paradigm shift in disease evaluation has been achieved, as global health initiatives are still rarely concerned with reduction in “burden of disease” besides mortality. This is also applicable to health planning at country or community level. In fact, the significance of noncommunicable/chronic health conditions such as cardiovascular diseases, cancer, diabetes, and mental illness is still being predominantly promoted on the basis of case fatality rather than disease burden. Conceptually, DALY and its variants like quality-adjusted life years (QALYs) represent equitable measures of population health that should be more actively promoted, but more work is required to address some of the methodological and equity concerns that have been raised since their introduction to global health [6,7].

The continued ranking of childhood diseases alongside adult diseases in the GBD report often portrays children as “young adults”. This practice distracts from diseases and conditions that have significant impacts on optimal early childhood development. Moreover, the risk of death for children younger than five years is projected to fall by more than 40% between 2005 and 2030, while life expectancy globally is expected to improve significantly by 2030, with the largest increases occurring in Africa and South Asia [1]. This trend is likely to bring to the fore the neglected discourse on the quality of life for the many survivors of acute childhood illnesses, particularly as the years lived with disability for chronic diseases of childhood onset far exceed those of adult onset. For instance, it is difficult to justify the continued failure to address a highly preventable perinatal condition such as neonatal jaundice, which may not be a leading cause of mortality but currently causes substantial burden in low-income countries [8].

Similarly, hearing loss of adult onset remains one of the ten leading causes of DALYs globally, particularly in high-income and middle-income countries, but is not expected to be a leading health problem in low-income countries by 2030 [1]. While this is gratifying to note, the current trend in the prevalence of disabling hearing loss (which more than doubled from 120 million in 1995 to about 278 million persons in 2005, two-thirds of whom reside in developing countries) may have much greater impact than projected, considering the envisaged improvement in life expectancy [9]. But more importantly, the exclusion of hearing loss of childhood onset or the failure to adequately account for permanent childhood hearing loss in these projections underrepresents the true burden of hearing loss. It disfranchises about 718,000 babies born annually in the developing world with neonatal hearing loss from available time-bound early detection and intervention services now a standard of neonatal care in developed countries [10].

The call by Mathers and Loncar for more robust studies across the regions of the world must not be overlooked or considered lightly. As new evidence emerges from such studies, the projected trend and ranking in many countries may ultimately differ from the overall global and regional picture in the current GBD report. Meanwhile, systematic steps to address these and other concerns should be considered urgently to effectively promote the concept of the burden of disease. Invariably, we all need to be guided by a variety of sources and perspectives in determining “the right stuff” for the heterogeneous populations of the world.
